# Chronic lymphocytic leukaemia induces an exhausted T cell phenotype in the TCL1 transgenic mouse model

**DOI:** 10.1111/bjh.13467

**Published:** 2015-05-04

**Authors:** Franz J Gassner, Nadja Zaborsky, Kemal Catakovic, Stefan Rebhandl, Michael Huemer, Alexander Egle, Tanja N Hartmann, Richard Greil, Roland Geisberger

**Affiliations:** 1 Laboratory for Immunological and Molecular Cancer Research, 3^rd^ Medical Department with Haematology, Medical Oncology, Haemostaseology, Infectiology and Rheumatology, Oncologic Centre, Paracelsus Medical UniversitySalzburg, Austria; 2 Salzburg Cancer Research InstituteSalzburg, Austria

**Keywords:** chronic lymphocytic leukaemia, tumour surveillance, T cell exhaustion, PDCD1, chemoimmunotherapy

## Abstract

Although chronic lymphocytic leukaemia (CLL) is a B cell malignancy, earlier studies have indicated a role of T cells in tumour growth and disease progression. In particular, the functional silencing of antigen-experienced T cells, called T cell exhaustion, has become implicated in immune evasion in CLL. In this study, we tested whether T cell exhaustion is recapitulated in the TCL1^tg^ mouse model for CLL. We show that T cells express high levels of the inhibitory exhaustion markers programmed cell death 1 (PDCD1, also termed PD-1) and lymphocyte-activation gene 3 (LAG3), whereas CLL cells express high levels of CD274 (also termed PD-ligand 1). In addition, the fraction of exhausted T cells increases with CLL progression. Finally, we demonstrate that exhausted T cells are reinvigorated towards CLL cytotoxicity by inhibition of PDCD1/CD274 interaction *in vivo*.

These results suggest that T cell exhaustion contributes to CLL pathogenesis and that interference with PDCD1/CD274 signalling holds high potential for therapeutic approaches.

Persistent exposure to antigen, as is common in chronic infections and during tumour outgrowth, induces a state of progressive T cell dysfunction known as T cell exhaustion. Exhausted T cells are characterized by poor effector function and the continued expression of inhibitory receptors (Wherry, 2011), of which co-expression of the inhibitory receptors programmed cell death 1 (PDCD1, also termed PD-1) and lymphocyte-activation gene 3 (LAG3) was most typical (Blackburn *et al*, 2009).

During T cell receptor (TCR) signalling, PDCD1 binds to its ligands CD274 (also termed PD-L1) and PDCD1LG2 (also termed PD-L2) (PD-Ls) and mediates the dephosphorylation of signalling molecules downstream of the T cell receptor, leading to a reduced signalling through ZAP70, CD3ζ and PI3K and, consequently, to decreased sensitivity to antigenic stimulation (Baitsch *et al*, 2012). Thus, PDCD1 and its ligands serve as important negative regulators of immune responses. In T cell exhaustion, the PDCD1 axis is thought to be the main inhibitory receptor pathway, contributing to the functional deficiencies observed in exhausted cells, including reduced cytokine production and decreased proliferative potential (Schietinger & Greenberg, 2014).

LAG3 is an activation-induced surface molecule that binds to major histocompatibility complex (MHC) class II with high affinity. It plays an important role in negatively regulating both the expansion of activated primary T cells and the development of the memory T cell pool (Workman *et al*, 2004) and, together with PDCD1, synergistically inhibits T cell functions to promote tumour immune escape (Woo *et al*, 2012).

Chronic lymphocytic leukaemia (CLL) is characterized by the accumulation of CD5^+^ B cells in the peripheral blood and lymphoid organs of patients, and a key role of the microenvironment in the pathogenesis of this disease is increasingly being understood (reviewed in (Pleyer *et al*, 2009)). Because the increase in tumour burden occurs over a long period of time, we hypothesized that the continued exposure of T cells to a CLL-associated antigen could lead to a state of T cell exhaustion. Indeed, T cell dysfunction occurring alongside CLL is well documented (reviewed in Hamblin & Hamblin, 2008), and includes an increase in absolute CD4^+^ and CD8^+^ T cell numbers, inversion of CD4/CD8 T cell ratio (Mackus *et al*, 2003), increase in death receptor expression (Tinhofer *et al*, 1998), loss of costimulatory molecules necessary for stimulation of antigen-presenting cells (APCs) (Frydecka *et al*, 2004), abnormal cytokine/receptor profile (Scrivener *et al*, 2003) and impaired formation of the immune synapse (Ramsay *et al*, 2008). Recently it was reported that T cells isolated from CLL patients express higher levels of PDCD1 than those from healthy donors and that the inversed CD4/CD8 T cell ratio in this disease is associated with a replicative senescence CD8^+^ phenotype (Nunes *et al*, 2012). These data suggest a CLL-induced expansion of exhausted CD8^+^ T cells with reduced anti-tumour activity. Concomitantly, Brusa *et al* (2013) found that blocking PDCD1/PD-L interactions *in vitro* leads to the production of interferon-γ in cytotoxic T lymphocytes (CTL) from CLL patients. In line with that, Ramsay *et al* (2012) showed increased PDCD1 expression on CLL T cells as well as increased CD274 expression on CLL tumour cells, which can be reduced by lenalidomide treatment, leading to increased CLL-T cell synapse formation. Our own studies corroborated these results as we observed that lenalidomide decreases the expression of inhibitory exhaustion markers on T cells (Gassner *et al*, 2014). These recent findings strongly suggest a fundamental contribution of the PDCD1/CD274 pathway to CLL tumour escape strategies. However, *in vivo* reinvigoration of T cells in CLL by blocking this pathway has not been reported yet. We therefore investigated whether T cell exhaustion is induced in the Eμ-TCL1 transgenic (TCL1^tg^) mouse model for CLL (Bichi *et al*, 2002), which recapitulates many of the features of human CLL, including the abnormalities in the T cell compartment (Gorgun *et al*, 2009; Hofbauer *et al*, 2011). Our results show that TCL1^tg^ mice exhibit features of T cell exhaustion similar to human CLL. In addition, we demonstrate that blockade of the PDCD1/CD274 pathway leads to increased tumour cell clearance in the TCL1^tg^ mouse model and might hence represent a promising new option in treatment of this disease.

## Material and methods

### Murine TCL1^tg^ tumour model and tumour transplantation model

All animal experiments were performed under approval from the central Austrian animal ethics committee. The original Eμ-TCL1 transgenic (TCL1^tg^) mice (Carlo M Croce, Department of Molecular Virology, Immunology and Medical Genetics, Ohio State University OH, USA) were backcrossed for >10 generations to C57BL/6J mice. These mice develop a clonal CD5^+^ B cell leukaemia manifested by peripheral-blood lymphocytosis and splenomegaly at a median of 11 months (Bichi *et al*, 2002; Johnson *et al*, 2006; Holler *et al*, 2009). Transplants were performed by injection of 2 × 10^7^ splenocytes of animals with established CLL disease into the peritoneal cavity of 6- to 8-week-old non-irradiated C57BL/6J WT mice. In the transplanted mice, the onset of disease is significantly shortened to a median latency period of 3 months (Hofbauer *et al*, 2011). Mice were followed for signs of illness and sacrificed when moribund (increased abdominal size, lethargy, decreased motion), and samples of peripheral blood, axillary and inguinal lymph nodes and spleen were collected for flow cytometric analysis.

### Flow cytometry analysis

Purified murine peripheral blood mononuclear cells or tail vein blood were incubated with fluorochrome-labelled rat anti-mouse antibodies directed to the following antigens were used: PDCD1 [phycoerythrin (PE), clone RPM1-30], LAG3 (PE, clone C9B7W), CD274 (PE, clone 10F.9G2) or the appropriate isotype controls, CD3 (fluorescein isothiocyanate, FITC), CD5 (PE-cyanin 5.1, PC5), CD8 (Biotin), CD19 (FITC), (all from Biolegend, San Diego, CA, USA) and streptavidin (Texas Red) (BD Biosciences, San Jose, CA, USA). Erythrocytes were lysed using BD fluorescence-activated cell sorting (FACS) Lysing solution (BD Biosciences). Cells were then resuspended in phosphate-buffered saline and analysed using an FC500 or Gallios flow cytometer and CXP1.0 software or Gallios Cytometer Software 1.1 (Beckman Coulter, Indianapolis, IN, USA). Results are calculated as percent positive cells (PDCD1 and LAG3) or mean fluorescence intensity ratio (MFIR; CD274 vs isotype control) as indicated.

### Mouse tumour-specific cytotoxicity assays

To describe the *in vivo* killing of tumour target cells by reinvigorated specific CTL, we assumed that at least a portion of exhausted T cells found in tumour transplanted mice are specific for the TCL1^tg^ tumour. To test whether exhausted T cells in these mice can be reactivated by inhibition of the PDCD1/PD-L pathway, we injected a mixture of differentially labelled tumour cells [carboxyfluorescein succinimidyl ester (CFSE)- and CellTrace™ violet-labelled target cells, Life Technologies, Carlsbad, CA, USA] into tail veins of these tumour transplanted mice. Prior to tail vein injection, CD274 was blocked on cells stained with CellTrace™ violet using recombinant PDCD1 (rPDCD1, amino acid 22-170, ProSci-Inc, Poway, CA, USA) or anti-CD274 F(ab) fragments [papain digestion of rat-anti-mouse CD274, clone MIH6, AbD Serotech (Kidlington, UK), according to manual] as indicated, while CFSE-labelled target cells were left untreated. Both target cells were mixed and injected into tumour-transplanted mice. Selective killing of target cells was monitored in recipient mice at 2 h and 24 h after transfer in peripheral blood as well as 24 h after transfer in various organs. A detailed assay overview is given in Fig S1.

### Statistical analysis

All statistical analyses were performed using SPSS Statistics 20 (IBM Armonk, NY, USA) or Graph Pad Prism 5. Boxplots show median (horizontal line in box), difference between 25th and 75th percentile (length of box) and data range (whiskers) unless stated otherwise. Where normally distributed, paired or unpaired student's *t*-tests were used for comparison between groups. For non-parametric comparisons between groups the Wilcoxon and Mann–Whitney *U* tests were used for paired and unpaired values, respectively. Values reported in the results section are in mean ± standard deviation and *P* value (paired or unpaired student's *t*-test) unless stated otherwise. A *P* value of 0·05 was used to define statistical significance. Graphs were created using GraphPad Prism 5 (GraphPad Software Inc, La Jolla, CA, USA).

## Results

### PDCD1 and LAG3 expression are increased in T cells of leukaemic mice

**Fig. 1:**
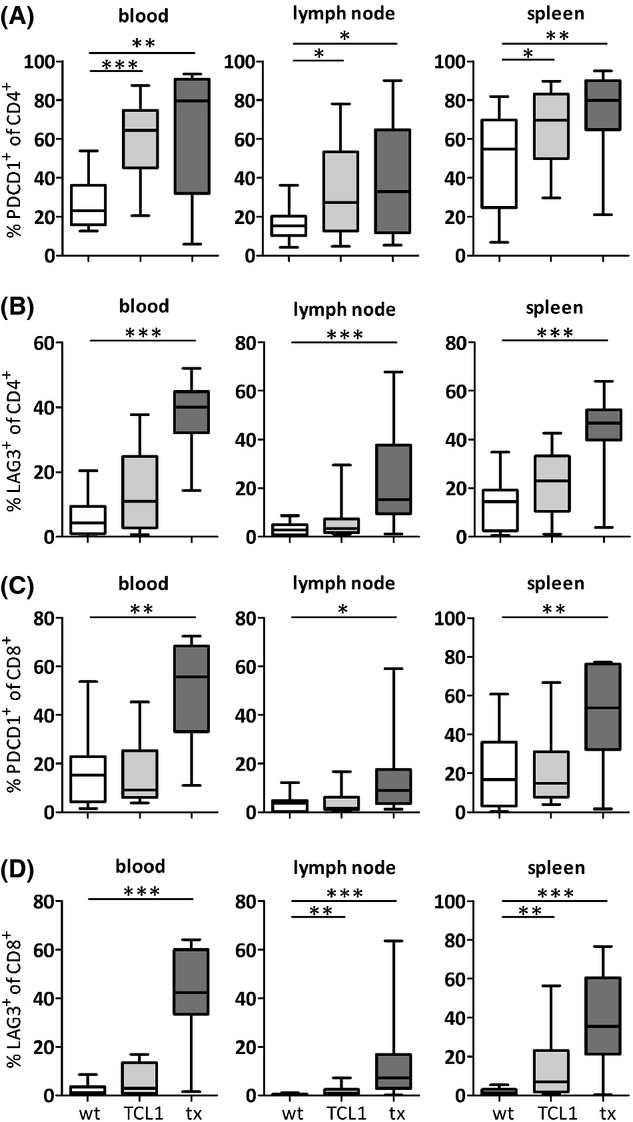
Wild-type mice (wt; *n* = 15; white), TCL1^tg^ mice (tcl1; *n* = 13; light grey) or tumour transplanted mice (tx; *n* = 15; dark grey) were sacrificed and peripheral blood, lymph node or spleen cells were analysed on a flow cytometer for the percentage of PDCD1 or LAG3 expressing CD4^+^ (A, B) or CD8^+^ (C, D) T cells. Bars indicate median. *P* values *<0·05, **<0·01 and ***<0·001 were considered significant.

We evaluated the expression of the exhaustion markers PDCD1 and LAG3 on T cells of spleen, lymph nodes and peripheral blood of sick TCL1^tg^ mice with established CLL (the mean percentage of CD19^+^ CD5^+^ malignant B cells in the peripheral blood was 60·0% ± 33·3%). The number of PDCD1-expressing cells was significantly increased in the CD4^+^ T cell compartment in the peripheral blood, axillary and inguinal lymph nodes and spleen of TCL1^tg^ mice as compared to wildtype (WT) age-matched control mice (Fig1A), while the number of LAG3^+^ CD4^+^ T cells was comparable (Fig1B). In the CD8^+^ population, there was no difference in the number of PDCD1^+^ cells (Fig1C) in any of the compartments examined, however the number of LAG3^+^ CD8^+^ T cells was significantly increased in lymph nodes and spleen (Fig1D). Because exhausted T cells in mice accumulate with age (Shimatani *et al*, 2009), we determined if the increase in the number of PDCD1 expressing T cells correlated with age in TCL1^tg^ mice. Notably, increased levels of PDCD1 expressing CD4^+^ T cells in the TCL1^tg^ mice were already discernable at the age of 200 days and no further increase was noticed during the leukaemic phase of the disease (Fig S2). In addition to primary TCL1^tg^ animals, we also utilized our recently established tumour-transplantation model, that leads to an accelerated tumour growth (Hofbauer *et al*, 2011) to clearly demonstrate a cause-and-effect relationship between the presence of CLL cells and the appearance of recipient T cells with an exhausted phenotype. WT mice transplanted with CLL were sacrificed when overt clinical symptoms of leukaemia developed at a mean age of 5·2 (range 2·7–9·4) months. As shown in Fig1, the number of PDCD1^+^ and LAG3^+^ T cells was increased in both CD4^+^ and CD8^+^ populations and in all lymphoid compartments examined of the recipient mice, but not in recipient mice receiving WT splenocytes (Fig S3). Furthermore, expression of PDCD1 and LAG3 was highly correlating in all compartments (Fig S4).

### CD274 is upregulated on murine CLL cells in lymphoid compartments

**Fig. 2:**
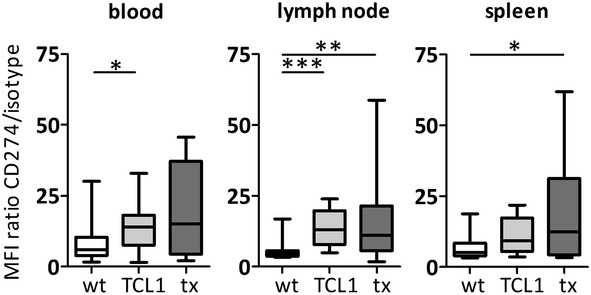
Expression of CD274 on B cells from wild-type (wt; *n* = 14; white), or CD19^+^ CD5^+^ tumour cells from TCL1^tg^ mice (tcl1; *n* = 12; light grey) or tumour transplanted mice (tx; *n* = 11; dark grey). Scale depicts mean fluorescence intensity (MFI) ratios of CD274 to isotype control antibody. Bars indicate median. *P* values *<0·05, **<0·01 and ***<0·001 were considered significant.

We next assessed the expression of the ligands for PDCD1 on the surface of tumour cells in mice. It has already been shown that CD274 is constitutively expressed at low levels on healthy splenic murine B cells (Yamazaki *et al*, 2002) and we confirmed these data in our WT mouse cohort (Fig2). Peripheral CLL tumour cells in TCL1^tg^ mice showed a modest increase in CD274 expression (MFIR 13·8 ± 8·0) as compared to WT B cells (MFIR 8·5 ± 7·5, *P* = 0·022), while lymph node-residing tumour cells exhibited a more pronounced increase (MFIR 14·0 ± 6·8 vs. 5·6 ± 3·3, *P* < 0·001), which suggests a microenvironment-induced upregulation of CD274 on tumour cells. When tumour-transplanted mice were sacrificed due to overt CLL, we again observed a trend for increased CD274 levels, which was most prominent on lymph node-residing CLL cells (Fig2; MFIR 17·2 ± 17·1, *P* = 0·003). Notably, we did not detect surface expression of PDCD1LG2 on any of the analysed samples (data not shown).

### Expression of T cell exhaustion markers correlates with CLL tumour cell infiltration in murine lymph nodes

**Fig. 3:**
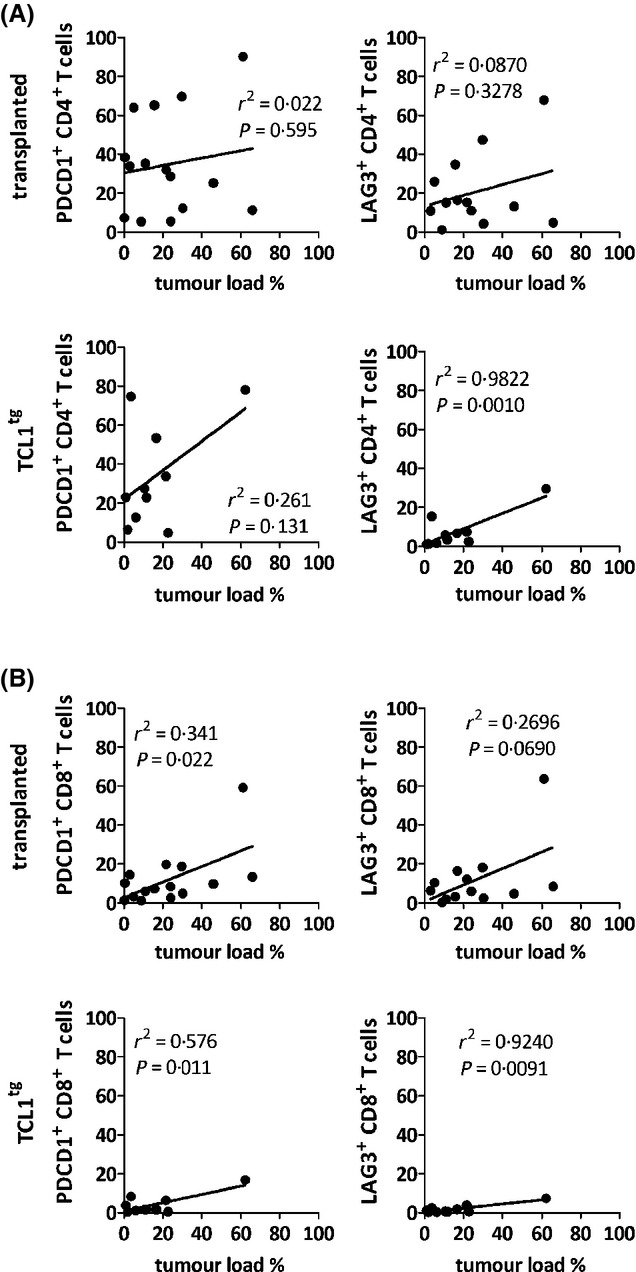
Correlation of organ tumour load with % CD4^+^ (A) or CD8^+^ (B) T cells expressing PDCD1 or LAG3 in lymph nodes of tumour transplanted or TCL1^tg^ mice.

As CLL cells could directly trigger the appearance of T cells exhibiting an exhausted phenotype, we analysed if the number of PDCD1-expressing T cells correlated with the tumour volume in each of the lymphoid organs examined. In lymph nodes of the TCL1^tg^ mice, a significant correlation between PDCD1 or LAG3 expression on T cells and tumour infiltration could be observed (Fig3), while there was no such correlation in the peripheral blood or spleen (not shown).

### PD-L inhibition leads to increased tumour lysis in mice

**Fig. 4:**
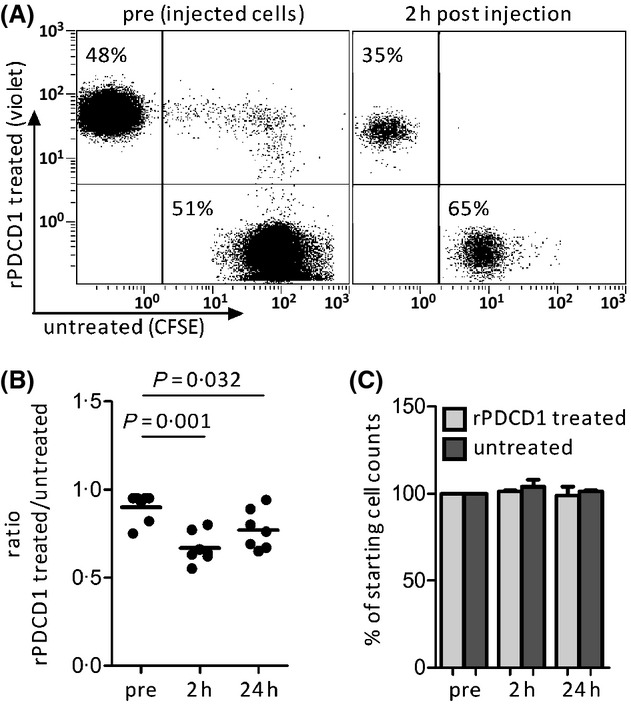
(A) Representative flow cytometric profiles of labelled and rPDCD1-treated (violet) *versus* untreated (CFSE) mouse tumour cells before (pre) and after tail vein injection into tumour bearing mice (2 h post-injection). Relative percentages of CFSE or CellTrace™ violet labelled chronic lymphocytic leuakaemia (CLL) cells are indicated in the quadrants of each plot. Values from 7 independent experiments are depicted in (B) as the ratio of rPDCD1-treated to untreated injected CLL cells (paired student's *t*-test). (C) Mouse tumour cells were treated with rPDCD1 *in vitro* or remained untreated, cultured in RPMI medium and cell numbers were determined at indicated time points (*n* = 2).

We speculated that the PDCD1/PD-L pathway might be exploited by CLL to evade a T cell-mediated cytotoxic attack and hence chose to utilize our murine tumour transplantation model to examine whether blocking the PDCD1/PD-L pathway by recombinant PDCD1 (rPDCD1) or anti-CD274 F(ab) fragments *in vivo* would lead to tumour lysis. In order to measure tumour killing by reinvigorated exhausted T cells, mice were first engrafted with a TCL1^tg^ tumour. When these mice became leukaemic, target cells (syngeneic TCL1^tg^ tumours) were adoptively transferred into these mice. Target cells were either untreated (which still have functional PD-L surface expression) or were incubated with rPDCD1 or anti-CD274 F(ab) fragments to block PD-L/PDCD1 interactions. These two target cell fractions were distinguished by fluorescently labelling them with different levels of CellTrace™ violet and CFSE fluorescent dyes. Mixtures of these cells were injected intravenously into TCL1^tg^ leukaemic recipient mice and the kinetics of the target cell ratios were analysed at 2 h and 24 h post-transfer in peripheral blood as well as 24 h post-transfer in spleen, lymph nodes and lung (assay outlined in Fig S1). We found that the fraction of the rPDCD1 treated tumour cells in peripheral blood was significantly reduced (2 h and 24 h after transfer) as compared to that of the untreated CLL cell fraction (Fig4A, B). Concomitantly, we found a reduction of CD274 blocked target cells also in other organs 24 h after transfer (Fig S5). Importantly, *in vitro* treatment with rPDCD1 fragment did not affect the viability of mouse tumour cells (Fig4C), implying that in tumour bearing mice, a vast number of tumour-specific exhausted CTLs exist that can be immediately reactivated by PDCD1/PD-L blockage, leading to cytolysis of the tumour.

## Discussion

Although autologous anti-tumour T cell responses in CLL can be induced upon *in vitro*-reactivation of T cells (Goddard *et al*, 2001), *in vivo* T cell cytotoxicity towards the malignant clone is vastly impaired, contributing to uncontrolled tumour growth. Here, we propose that a main cause for the lack of efficient T cell-mediated anti-tumour cytotoxicity *in vivo* is exhaustion of T cells specific for the CLL B cell clone, and we present several lines of evidence to support this. We show that T cells, especially in the murine CLL transplantation model, show increased percentages of PDCD1 and LAG3 expression, surface markers that are associated with an exhausted phenotype. This T cell phenotype is clearly induced by the presence of malignant B cells, because it rapidly develops upon tumour cell transplantation into WT mice but not upon transplantation with healthy splenocytes. Importantly, we see a positive correlation of tumour load and the expression of exhaustion markers on cytotoxic CD8^+^ T cells in lymph nodes in the tumour-bearing mice. This finding is particularly interesting, as the TCL1^tg^ mouse model is the only CLL mouse model to date that shows tumour infiltration in lymph nodes and where intense CLL-T cell interaction in this organ is suggested (Hofbauer *et al*, 2011). We therefore speculate that increased exhaustion might be the result of increased tumour-antigen exposure to tumour-specific T cells in specialized microenvironments. In line with that, while peripheral murine B and CLL cells constitutively express low levels of CD274, we detected increased CD274 levels on lymph node- or spleen-residing tumour cells. In a microenvironment where CLL cells are in tight contact with possible tumour-specific T cells this might serve as protection against cytotoxic attacks. Indeed, we found that in mice bearing high tumour load and a high number of circulating PDCD1^+^ CTLs, blocking the PDCD1/PD-L inhibitory pathway resulted in enhanced clearance of the tumour cells. First of all, this finding suggests that the PDCD1^+^ CTLs in these mice are indeed functionally exhausted and contain tumour-specific clones that can be reinvigorated by inhibiting PDCD1 ligation. Secondly, it shows that CLL cells are in fact vulnerable to cytolytic attacks when deprived of CD274-mediated protection. These data fit very well to previous results reported on human CLL, where PDCD1 expressing T cells were found in close contact with CD274 expressing CLL cells in proliferation centres (Brusa *et al*, 2013), and the exhausted phenotype of these T cells was reversible by blocking PDCD1/PD-L interactions or by downmodulation of PDCD1 by immune-modulatory drugs (Brusa *et al*, 2013; Ramsay *et al*, 2012).

Previous experiments on knockout mice revealed that the PDCD1/CD274 system has most likely evolved as means to ensure immunological tolerance to self-tissue, as mice deficient in PDCD1 suffer from spontaneous autoimmune diseases (Nishimura *et al*, 1998, 1999). More recently published work has shown that PDCD1 is highly expressed on CD8^+^ T cells during chronic lymphocytic choriomeningitis virus (LCMV) infection and that the PDCD1/PD-L pathway plays a major role for virus persistence by inhibiting an effective anti-virus response (Barber *et al*, 2006). Several consecutive studies have confirmed that the PDCD1/PD-L pathway is exploited by a panel of viral and parasitic infections as target for immune evasion, mostly by increasing CD274 on APCs (Kirchberger *et al*, 2005; Smith *et al*, 2004). The observation that PD-L is also expressed on some solid tumours and that blocking PDCD1/PD-L interaction actually accelerates tumour eradication in mouse models *in vivo* has pointed to involvement of PDCD1/PD-L in the suppression of anti-tumour immune responses and opened new doors for therapeutic intervention in cancer (Iwai *et al*, 2002; Curiel *et al*, 2003). In fact, a combination of cyclophosphamide and PDCD1 antibody resulted in synergistic augmentation of anti-tumour response in a murine tumour vaccination study (Mkrtichyan *et al*, 2011). We have previously shown that fludarabine, the standard component in modern CLL combinational treatment, modulates the T cell pool towards an antigen-experienced memory phenotype with increased proliferative potential (Gassner *et al*, 2011). Hence, following reduction of the bulk tumour by conventional chemotherapy, blocking the PDCD1 inhibitory pathway might release a ‘brake’ on the T cell receptor signalling and reasonably increase tumour-specific cytotoxicity. Inhibition of the PDCD1/PD-L pathway has already been shown to have substantial therapeutic efficacy and a good safety profile in relapsed or refractory Hodgkin Lymphoma (Ansell *et al*, 2015), advanced melanoma (Wolchok *et al*, 2013) and other advanced cancers, including non–small-cell lung cancer and renal-cell cancer (Brahmer *et al*, 2012), and an inhibitory PDCD1 antibody has recently gained US Food and Drug Administration approval for patients with advanced or unresectable melanoma (Momtaz & Postow, 2014). However, no data on PDCD1 blockade in CLL are available yet, as a phase II study of anti-PDCD1 treatment in relapsed or refractory CLL has just been initiated (ClinicalTrials.gov identifier: NCT02332980).

Here, we propose that the abundant presence of tumour antigen as well as tight CLL-T cell interactions in lymphoid tissues induce expression of PDCD1 on T cells. Lymph node-residing CLL cells, which have been shown to comprise activated, proliferating tumour cells with high expression of costimulatory and adhesion molecules (Patten *et al*, 2008; Plander *et al*, 2009), would thus be protected from T cell-mediated cytolysis by expressing CD274. Manipulating this protective mechanism by blocking the PDCD1/CD274 interaction may represent a promising target for therapeutic intervention, especially in combination with already established immunomodulatory or cytoreductive treatment options.

## Supporting Information

**Fig S1.** The experimental design of the mouse tumour specific cytotoxicity assays. **Fig S2.** Correlation of age with % CD4^+^ (A) or CD8^+^ (B) T cells expressing PDCD1 in blood, spleen and lymph node of wildtype or TCL1tg mice. **Fig S3.** Wildtype mice were either not transplanted (-; closed circles) or transplanted with splenocytes from wildtype (wt; closed squares) orTCLl1tg (TCL1; closed triangles) mice and analyzed 60 days after transplantation for expression of PDCD1 and LAG3 on CD4 + (A) or CD8 + (B) T cells in blood, lymph node and spleen. **Fig S4.** Correlation of Lag-3 and PD-1 expression on CD4^+^ (A) or CD8^+^ (B) T cells in blood, lymph node and spleen of wildtype or TCL1tg or tumour transplanted mice. **Fig S5.** The ratio of CD274 blocked [recombinant PDCD1 (A) or anti-CD274- F(ab) (B)] to unblocked target cells at injection, 2 h and 24 h after transfer.
