# Supplementary material for: Chronic lymphocytic leukaemia induces an exhausted T cell phenotype in the TCL1 transgenic mouse model
Source: Br J Haematol. 2015 May 4;170(4):515–22. doi: 10.1111/bjh.13467 (PMC4687418; doi:10.1111/bjh.13467)
Supplement: Supplementary file 1 [file bjh0170-0515-sd1.pdf]

Supporting Information S1

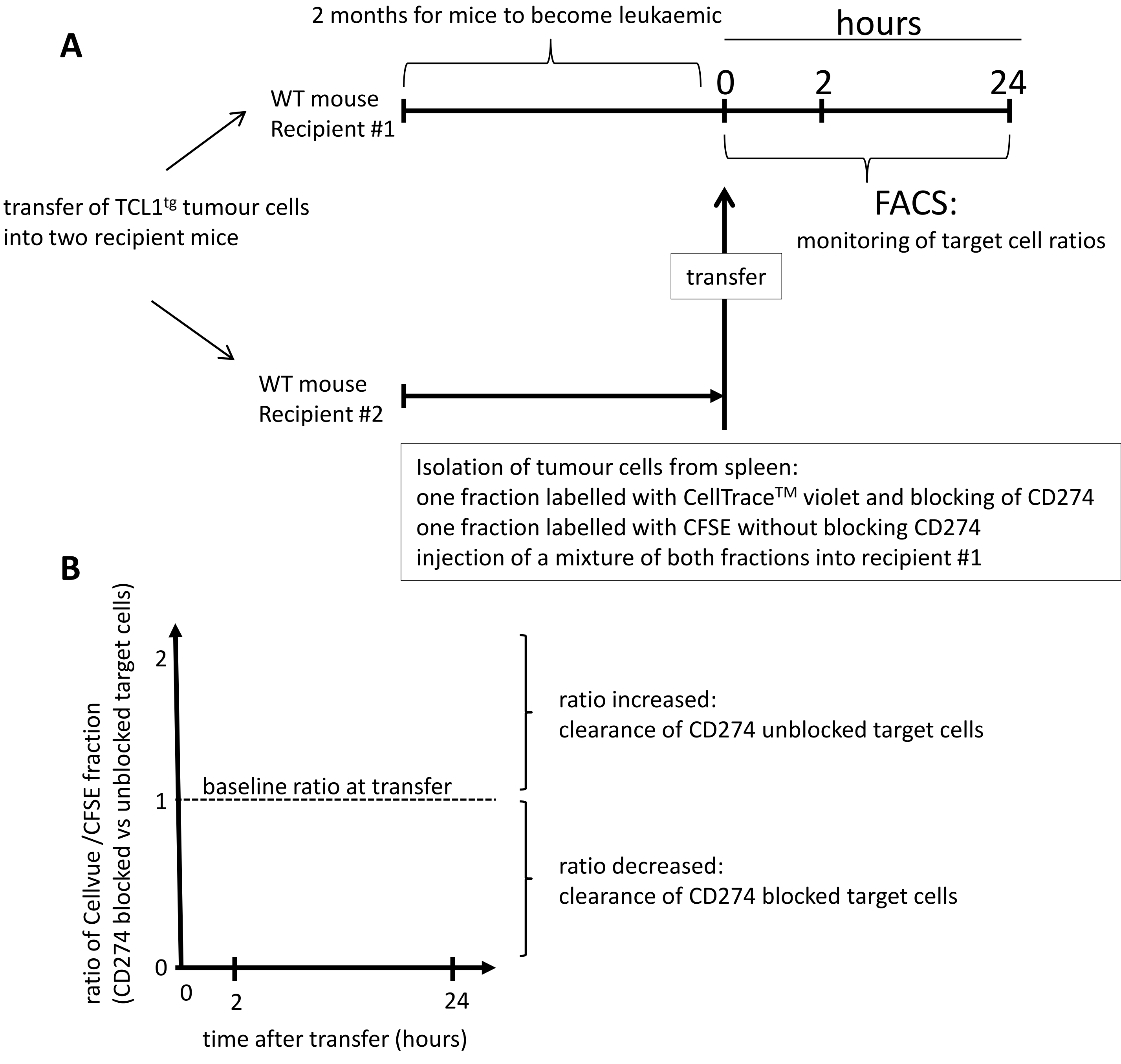

Supporting Information S1: The experimental design of the mouse tumour specific cytotoxicity assays. (***A***) A primary TCL1^{tg} tumour was injected into two WT recipient mice. In both mice, tumours were grown until a clear tumour load was visible in peripheral blood. The tumour from recipient #2 was isolated and treated as indicated in the figure followed by tail vein injection into recipient #1. After transfer, injected target cells were monitored in recipient #1 by flow cytometry. **(*B*)** The ratio of CD274 blocked and unblocked target cells was monitored by flow cytometry 2h and 24h after transfer. Changes in the ratio are interpreted as selective killing of either CD274 blocked (decreased ratio) or CD274 proficient (increased ratio) target cells.

Supporting Information S2

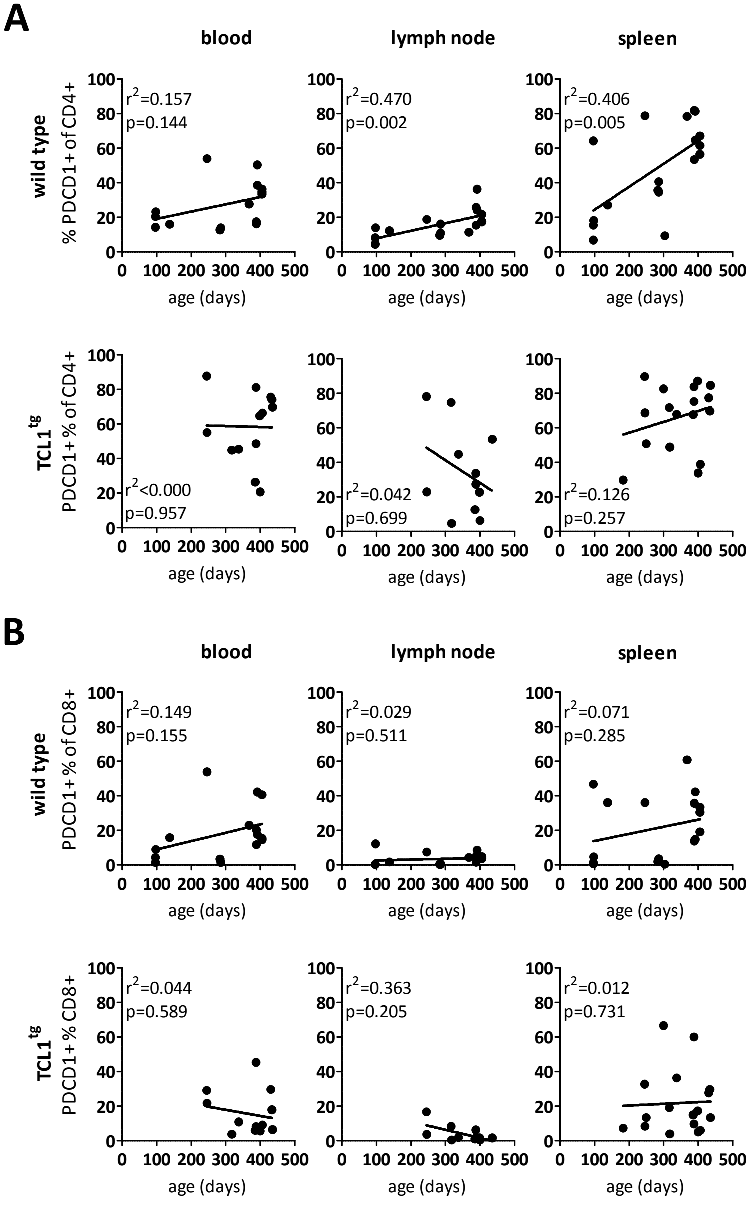

Supporting Information S2
Correlation of age with % CD4+ (A) or CD8+ (B) T cells expressing PDCD1 in blood, spleen and lymph node of wildtype or $TCL1^{tg}$ mice.

Supporting Information S3

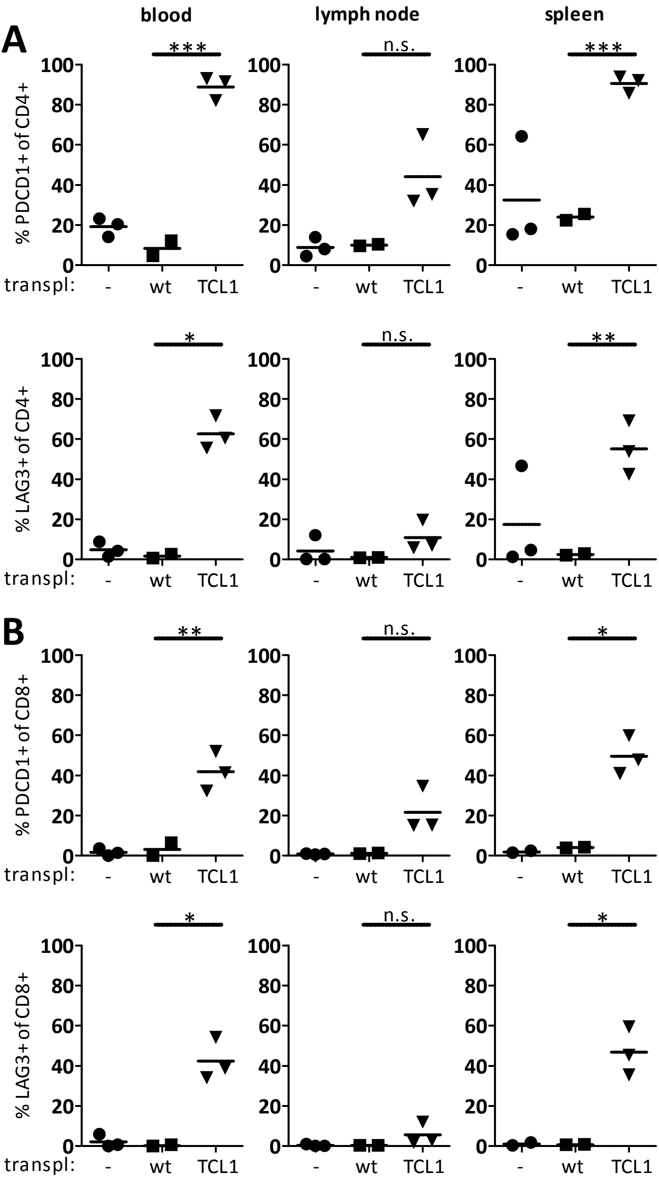

Supporting Information S3
Wildtype mice were either not transplanted (-; closed circles) or transplanted with splenocytes from wildtype (wt; closed squares) orTCLl1^{tg} (TCL1; closed triangles) mice and analyzed 60 days after transplantation for expression of PDCD1 and LAG3 on CD4+ (A) or CD8+ (B) T cells in blood, lymph node and spleen. All mice are age-matched. Significance was determined by unpaired student's t-test. *P* values *< 0.05, **< 0.01 and ***< 0.001 were considered significant. n.s.= not significant

Supporting Information S4

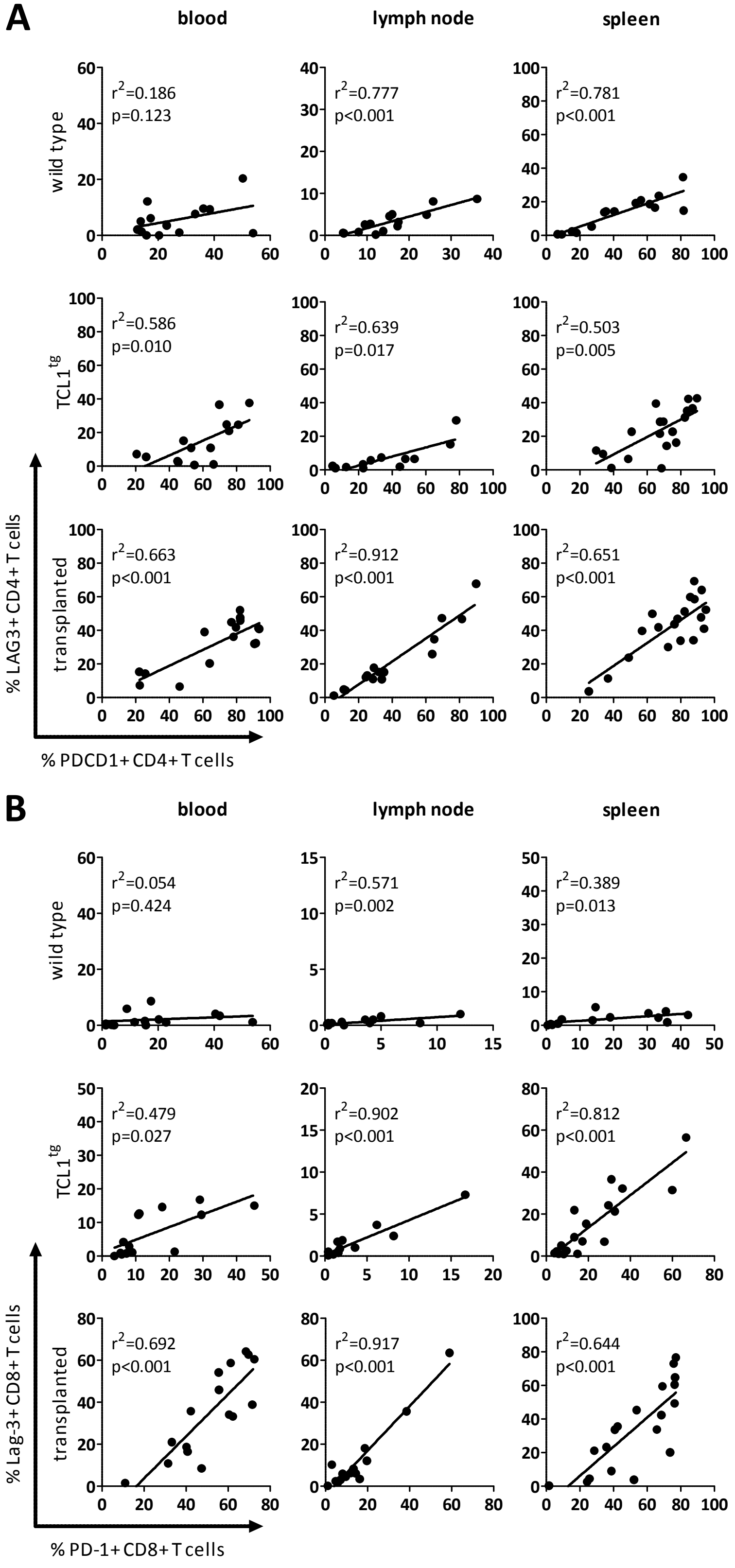

Supporting Information S4
Correlation of Lag-3 and PD-1 expression on CD4+ (A) or CD8+ (B) T cells in blood, lymph node and spleen of wildtype or TCL1^{tg} or tumour transplanted mice.

Supporting Information S5

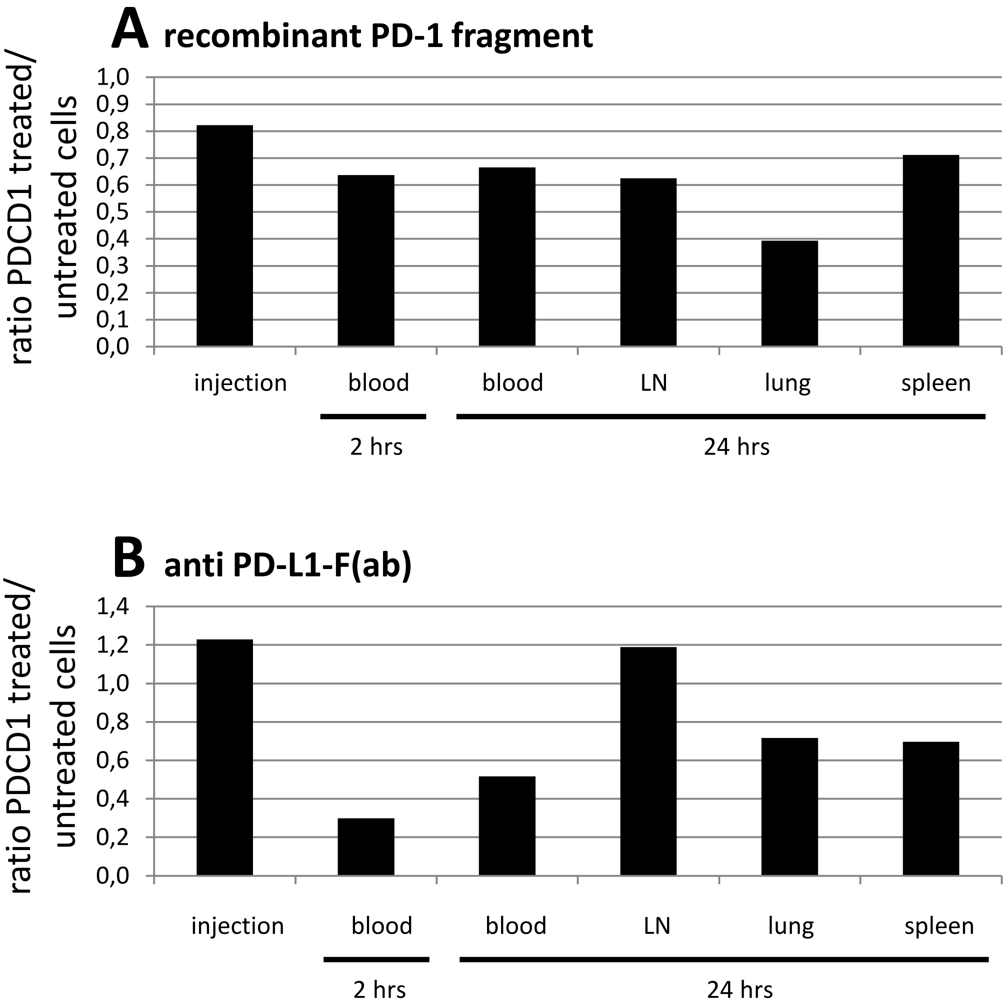

Supporting Information S5
The ratio of CD274 blocked (recombinant PDCD1 (A) or anti-CD274-F(ab) (B)) to unblocked target cells at injection, 2h and 24h after transfer. Changes in the ratio are interpreted as selective killing of either CD274 blocked (decreased ratio) or CD274 proficient (increased ratio) target cells. hrs, hours; LN, lymph node
